# Supplementary material for: Dynamic impact of E-WOM and green discourse on green hotel supply chain performance with time lag effect
Source: PLoS One. 2025 Jul 18;20(7):e0327324. doi: 10.1371/journal.pone.0327324 (PMC12274011; doi:10.1371/journal.pone.0327324)
Supplement: S1 File — (DOCX) [file pone.0327324.s001.docx]

**Supporting information**

**Proof of Proposition 1**

For $G^{O}$(t) > 0, $W^{O}$(t) > 0 the maximal profits of the GSMHE and the OTA at time t in the Hamilton-Jacobi-Bellman equation satisfy the following equations:

$$\begin{aligned} \rho V_{h}^{O}=\max_{S_{h}^{O}} \left\{ \begin{aligned} \pi_{h}\left( \alpha G^{O}\left( t \right)+\beta W^{O}\left( t \right) \right)-\frac{\mu_{1}}{\text{2}}{S_{h}^{O}}^{\text{2}}+\frac{\partial V_{h}^{O}}{\partial G^{O}}\left( \tau_{1}S_{h}^{O}\left( t \right)-\gamma G^{O}\left( t \right) \right) \\ +\frac{\partial V_{h}^{O}}{\partial W^{O}}\left( \eta G^{O}\left( t \right)+\tau_{3}E_{o}^{O}\left( t-d_{o} \right)-\delta W^{O}\left( t \right) \right) \end{aligned} \right\}\#\left( A.1 \right) \end{aligned}$$

$$\begin{aligned} \rho V_{o}^{O}=\max_{E_{o}^{O}} \left\{ \begin{aligned} \pi_{o}\left( \alpha G^{O}\left( t \right)+\beta W^{O}\left( t \right) \right)-\frac{\mu_{3}}{\text{2}}{E_{o}^{O}}^{\text{2}}+\frac{\partial V_{o}^{O}}{\partial G^{O}}\left( \tau_{1}S_{h}^{O}\left( t \right)-\gamma G^{O}\left( t \right) \right) \\ +\frac{\partial V_{o}^{O}}{\partial W^{O}}\left( \eta G^{O}\left( t \right)+\tau_{3}E_{o}^{O}\left( t-d_{o} \right)-\delta W^{O}\left( t \right) \right) \end{aligned} \right\}\#\left( A.2 \right) \end{aligned}$$

According to studies of Basin and Rodriguez-Gonzalez (2006) , Sun, et al. (2020) ,$\frac{\partial E_{o}(t-d_{o})}{\partial E_{o}(t)}=e^{\delta d_{o}}$.

From Eq. (A.1) and (A.2), we can obtain,

$$\begin{aligned} \left\{ \begin{aligned} S_{h}^{O*}&=\frac{\partial V_{h}^{O}}{\partial G^{O}}\frac{\tau_{1}}{\mu_{1}} \\ E_{o}^{O*}&=\frac{\partial V_{o}^{O}}{\partial W^{O}}\frac{\tau_{2}}{\mu_{2}}e^{\delta d_{o}} \end{aligned} \right.\#\left( A.3 \right) \end{aligned}$$

Substituting Eq. (A.3) into the Eq. (A.1) and Eq. (A.2), yields

$$\begin{aligned} \rho V_{h}^{O}=\left\{ \begin{aligned} &\pi_{h}\cdot\left( \alpha G^{O}\left( t \right)+\beta W^{O}\left( t \right) \right)-\frac{{\tau_{1}}^{2}}{\text{2}\mu_{1}}\left( \frac{\partial V_{h}^{O}}{\partial G^{O}} \right)^{\text{2}}+\frac{{\tau_{1}}^{2}}{\mu_{1}}\left( \frac{\partial V_{h}^{O}}{\partial G^{O}} \right)^{\text{2}}-\gamma{\frac{\partial V_{h}^{O}}{\partial G^{O}}G}^{O}\left( t \right) \\ &+\eta\frac{\partial V_{h}^{O}}{\partial W^{O}}G^{O}\left( t \right)+\frac{{\tau_{3}}^{2}}{\mu_{3}}\frac{\partial V_{h}^{O}}{\partial W^{O}}\frac{\partial V_{h}^{O}}{\partial W^{O}}e^{2\delta d_{o}}-\delta\frac{\partial V_{h}^{O}}{\partial W^{O}}W^{O}\left( t \right) \end{aligned} \right\}\#\left( A.4 \right) \end{aligned}$$

$$\begin{aligned} \rho V_{o}^{O}=\left\{ \begin{aligned} \pi_{o}&\cdot\left( \alpha G^{O}\left( t \right)+\beta W^{O}\left( t \right) \right)-\frac{{\tau_{3}}^{2}}{\text{2}\mu_{3}}\left( \frac{\text{∂}V_{o}^{O}}{\text{∂}\text{W}^{\text{O}}} \right)^{\text{2}}e^{2\delta d_{o}}+\frac{{\tau_{1}}^{2}}{\mu_{1}}\frac{\text{∂}V_{h}^{O}}{\text{∂}\text{G}^{\text{O}}}\frac{\text{∂}V_{o}^{O}}{\text{∂}\text{G}^{\text{O}}} \\ -\gamma&{\frac{\partial V_{o}^{O}}{\partial G^{O}}G}^{O}\left( t \right)+\eta\frac{\partial V_{o}^{O}}{\partial W^{O}}G^{O}\left( t \right)+\frac{{\tau_{3}}^{2}}{\mu_{3}}\left( \frac{\text{∂}V_{O}^{O}}{\text{∂}\text{W}^{\text{O}}} \right)^{\text{2}}e^{2\delta d_{o}}-\delta{\frac{\text{∂}V_{o}^{O}}{\text{∂}\text{W}^{\text{O}}}W}^{O}\left( t \right) \end{aligned} \right\}\#\left( A.5 \right) \end{aligned}$$

It can be seen from Eq. (A.4) and Eq. (A.5) that the solution of the HJB equation is a linear function of G(t), W(t) , then we set $V_{h}^{O}=n_{1}G^{O}\left( t \right)+n_{2}W^{O}\left( t \right)+n_{3}$, $V_{o}^{O}=n_{4}G^{O}\left( t \right)+n_{5}W^{O}\left( t \right)+n_{6}$where $n_{1}$,$n_{2}$,$n_{3}$,$n_{4}, n_{5}$,$n_{6}$are constants, $\frac{\partial V_{h}^{O}}{\partial G^{O}}=n_{1}$, $\frac{\partial V_{h}^{O}}{\partial W^{O}}=n_{2}$,$\frac{\text{∂}V_{o}^{O}}{\text{∂}\text{G}^{\text{O}}}=n_{4}$, $\frac{\partial V_{o}^{O}}{\partial W^{O}}= n_{5}$, then

$\begin{aligned} &\rho\left( n_{1}G^{O}\left( t \right)+n_{2}W^{O}\left( t \right)+n_{3} \right) \\ &= \left\{ \begin{aligned} \pi_{h}&\cdot\left( \alpha G^{O}\left( t \right)+\beta W^{O}\left( t \right) \right)-\frac{{\tau_{1}}^{2}}{\text{2}\mu_{1}}\left( \frac{\partial V_{h}^{O}}{\partial G^{O}} \right)^{\text{2}}+\frac{{\tau_{1}}^{2}}{\mu_{1}}\left( \frac{\partial V_{h}^{O}}{\partial G^{O}} \right)^{\text{2}}-\gamma{\frac{\partial V_{h}^{O}}{\partial G^{O}}G}^{O}\left( t \right) \\ +\eta&\frac{\partial V_{h}^{O}}{\partial W^{O}}G^{O}\left( t \right)+\frac{{\tau_{2}}^{2}}{\mu_{2}}\frac{\partial V_{h}^{O}}{\partial W^{O}}\frac{\partial V_{h}^{O}}{\partial W^{O}}e^{2\delta d_{o}}-\delta\frac{\partial V_{h}^{O}}{\partial W^{O}}W^{O}\left( t \right) \# \end{aligned} \right\}\# \left( A.6 \right) \end{aligned}$

$$\begin{aligned} &\rho\left( n_{4}G^{O}\left( t \right)+n_{5}W^{O}\left( t \right)+n_{6} \right) \\ &=\left\{ \begin{aligned} \pi_{o}&\cdot\left( \alpha G^{O}\left( t \right)+\beta W^{O}\left( t \right) \right)-\frac{{\tau_{3}}^{2}}{\text{2}\mu_{3}}\left( \frac{\text{∂}V_{o}^{O}}{\text{∂}\text{W}^{\text{O}}} \right)^{\text{2}}e^{2\delta d_{o}}+\frac{{\tau_{1}}^{2}}{\mu_{1}}\frac{\text{∂}V_{h}^{O}}{\text{∂}\text{G}^{\text{O}}}\frac{\text{∂}V_{o}^{O}}{\text{∂}\text{G}^{\text{O}}}-\gamma{\frac{\partial V_{o}^{O}}{\partial G^{O}}G}^{O}\left( t \right) \\ +\eta&\frac{\partial V_{o}^{O}}{\partial W^{O}}G^{O}\left( t \right)+\frac{{\tau_{3}}^{2}}{\mu_{3}}\left( \frac{\text{∂}V_{O}^{O}}{\text{∂}\text{W}^{\text{O}}} \right)^{\text{2}}e^{2\delta d_{o}}-\delta{\frac{\text{∂}V_{o}^{O}}{\text{∂}\text{W}^{\text{O}}}W}^{O}\left( t \right) \end{aligned} \right\}\#\left( A.7 \right) \end{aligned}$$

From Eq. (A.5) and (A.6), yields

$$\begin{aligned} \left\{ \begin{aligned} n_{1}&=\frac{\pi_{h}\left( \alpha\left( \rho+\delta\right)+\beta\eta\right)}{\left( \rho+\gamma\right)\left( \rho+\delta\right)} \\ n_{2}&=\frac{\beta\pi_{h}}{\left( \rho+\delta\right)} \\ n_{3}&=\frac{{\pi_{h}}^{2}\left( \alpha\left( \rho+\delta\right)+\beta\eta\right)^{2}}{2\rho{\left( \rho+\delta\right)^{2}\left( \rho+\gamma\right)}^{2}}\frac{{\tau_{1}}^{2}}{\mu_{1}}+\frac{\pi_{h}\pi_{o}\beta^{2}}{\rho({\rho+\delta)}^{2}}\frac{{\tau_{3}}^{2}}{\mu_{3}}e^{2\delta d_{o}} \end{aligned} \right.\#\left( A.8 \right) \end{aligned}$$

And

$$\begin{aligned} \left\{ \begin{aligned} n_{4}&=\frac{\pi_{o}\left( \alpha\left( \rho+\delta\right)+\beta\eta\right)}{\left( \rho+\gamma\right)\left( \rho+\delta\right)} \\ n_{5}&=\frac{\beta\pi_{o}}{\left( \rho+\delta\right)} \\ n_{6}&=\frac{{\pi_{o}}^{2}\beta^{2}}{2\rho({\rho+\delta)}^{2}}\frac{{\tau_{3}}^{2}}{\mu_{3}}e^{2\delta d_{o}}+\frac{\pi_{h}\pi_{o}\left( \alpha\left( \rho+\delta\right)+\beta\eta\right)^{2}}{\rho{\left( \rho+\delta\right)^{2}\left( \rho+\gamma\right)}^{2}}\frac{{\tau_{1}}^{2}}{\mu_{1}} \end{aligned} \right.\#\left( A.9 \right) \end{aligned}$$

Substituting, $\frac{\partial V_{h}^{O}}{\partial G^{O}}=n_{1}$, $\frac{\partial V_{h}^{O}}{\partial W^{O}}=n_{2}$, $n_{3}$ into Eq. (A.5) and $\frac{\text{∂}V_{o}^{O}}{\text{∂}\text{G}^{\text{O}}}=n_{4}$, $\frac{\text{∂}V_{o}^{O}}{\text{∂}\text{W}^{\text{O}}}=n_{5}$,$n_{6}$ into Eq. (A.3) . We can obtain Proposition 1.

**Proof of proposition 3**

For $G^{C}$(t) > 0, $W^{C}$(t) > 0 the maximal profits of the GSMHE and the OTA at time t in the Hamilton-Jacobi-Bellman equation satisfy the following equations:

$\begin{aligned} \rho V_{h}^{C}=\max_{S_{h}^{C},E_{h}^{C}} \left\{ \begin{aligned} \pi_{h}&\left( \alpha G^{C}\left( t \right)+\beta W^{C}\left( t \right) \right)-\frac{\mu_{1}}{\text{2}}{S_{h}^{C}}^{\text{2}}-\frac{\mu_{2}}{\text{2}}{E_{h}^{C}}^{\text{2}} \\ +&\frac{\partial V_{h}^{C}}{\partial G^{C}}\left( \tau_{1}S_{h}^{C}\left( t \right)-\gamma G^{C}\left( t \right) \right)+ \\ \frac{\partial V_{h}^{C}}{\partial W^{C}}&\left( \eta G^{C}\left( t \right)+\tau_{2}E_{o}^{C}\left( t-d_{h} \right)+\tau_{3}E_{o}^{C}\left( t-d_{o} \right)-\delta W^{C}\left( t \right) \right) \end{aligned} \right\}\#\left( A.10 \right) \end{aligned}$

$$\begin{aligned} \rho V_{o}^{C}=\max_{E_{o}^{C}} \left\{ \begin{aligned} \pi_{o}\left( \alpha G^{C}\left( t \right)+\beta W^{C}\left( t \right) \right)-\frac{\mu_{3}}{\text{2}}{E_{o}^{C}}^{\text{2}}+\frac{\partial V_{o}^{C}}{\partial G^{C}}\left( \tau_{1}S_{h}^{C}\left( t \right)-\gamma G^{C}\left( t \right) \right) \\ +\frac{\partial V_{o}^{C}}{\partial W^{C}}\left( \eta G^{C}\left( t \right)\tau_{2}E_{o}^{C}\left( t-d_{h} \right)+\tau_{3}E_{o}^{C}\left( t-d_{o} \right)-\delta W^{C}\left( t \right) \right) \end{aligned} \right\}\# \left( A.11 \right) \end{aligned}$$

According to studies of Basin and Rodriguez-Gonzalez (2006) , Sun, et al. (2020) ,$\frac{\partial E_{o}(t-d_{o})}{\partial E_{o}(t)}=e^{\delta d_{o}}$,$\frac{\partial E_{h}(t-d_{h})}{\partial E_{h}(t)}=e^{\delta d_{h}}$.

From Eq. (A.10) and (A.11), we can obtain:

$$\begin{aligned} \left\{ \begin{aligned} S_{h}^{C*}&=\frac{\partial V_{h}^{C}}{\partial G^{C}}\frac{\tau_{1}}{\mu_{1}} \\ E_{o}^{C*}&=\frac{\partial V_{h}^{C}}{\partial W^{C}}\frac{\tau_{2}}{\mu_{2}}e^{\delta d_{h}} \\ E_{o}^{C*}&=\frac{\partial V_{o}^{C}}{\partial W^{C}}\frac{\tau_{2}}{\mu_{2}}e^{\delta d_{o}} \end{aligned} \right.\#\left( A.12 \right) \end{aligned}$$

Substituting Eq. (A.12) into the Eq. (A.10) and (A.11), yields

$$\begin{aligned} \rho V_{h}^{C}=\left\{ \begin{aligned} \pi_{h}&\left( \alpha G^{C}\left( t \right)+\beta W^{C}\left( t \right) \right)-\frac{{\tau_{1}}^{2}}{\text{2}\mu_{1}}\left( \frac{\partial V_{h}^{C}}{\partial G^{C}} \right)^{\text{2}}-\frac{{\tau_{2}}^{2}}{\text{2}\mu_{2}}\left( \frac{\partial V_{h}^{C}}{\partial W^{C}} \right)^{\text{2}}e^{2\delta d_{h}} \\ +&\frac{{\tau_{1}}^{2}}{\mu_{1}}\left( \frac{\partial V_{h}^{C}}{\partial G^{C}} \right)^{\text{2}}-\gamma{\frac{\partial V_{h}^{C}}{\partial G^{C}}G}^{C}\left( t \right)+\eta\frac{\partial V_{h}^{C}}{\partial W^{C}}G^{C}\left( t \right) \\ +\frac{{\tau_{2}}^{2}}{\mu_{2}}\left( \frac{\partial V_{h}^{C}}{\partial W^{C}} \right)^{\text{2}}e^{2\delta d_{h}}+\frac{{\tau_{3}}^{2}}{\mu_{3}}\frac{\partial V_{h}^{C}}{\partial W^{C}}\frac{\partial V_{h}^{C}}{\partial W^{C}}e^{2\delta d_{o}}-\delta\frac{\partial V_{h}^{C}}{\partial W^{C}}W^{C}\left( t \right) \end{aligned} \right\}\#\left( A.13 \right) \end{aligned}$$

$$\begin{aligned} \rho V_{o}^{C}=\left\{ \begin{aligned} \pi_{o}&\left( \alpha G^{C}\left( t \right)+\beta W^{C}\left( t \right) \right)-\frac{{\tau_{3}}^{2}}{\text{2}\mu_{3}}\left( \frac{\text{∂}V_{o}^{C}}{\text{∂}\text{W}^{\text{C}}} \right)^{\text{2}}e^{2\delta d_{o}} \\ +&\frac{{\tau_{1}}^{2}}{\mu_{1}}\frac{\text{∂}V_{h}^{C}}{\text{∂}\text{G}^{\text{C}}}\frac{\text{∂}V_{o}^{C}}{\text{∂}\text{G}^{\text{C}}}-\gamma{\frac{\partial V_{o}^{C}}{\partial G^{C}}G}^{C}\left( t \right)+\eta\frac{\partial V_{o}^{C}}{\partial W^{C}}G^{C}\left( t \right) \\ +&\frac{{\tau_{2}}^{2}}{\mu_{2}}\frac{\partial V_{h}^{C}}{\partial W^{C}}\frac{\partial V_{o}^{C}}{\partial W^{C}}e^{2\delta d_{h}}+\frac{{\tau_{3}}^{2}}{\mu_{3}}\left( \frac{\text{∂}V_{O}^{C}}{\text{∂}\text{W}^{\text{C}}} \right)^{\text{2}}e^{2\delta d_{o}}-\delta{\frac{\text{∂}V_{o}^{C}}{\text{∂}\text{W}^{\text{C}}}W}^{C}\left( t \right) \end{aligned} \right\}\#\left( A.14 \right) \end{aligned}$$

It can be seen from Eq. (A.13) and (A.14) that the solution of the HJB equation is a linear function of G(t), W(t) , then we set $V_{h}^{C}=f_{1}G^{C}\left( t \right)+f_{2}W^{C}\left( t \right)+f_{3}$, $V_{o}^{C}=f_{4}G^{C}\left( t \right)+f_{5}W^{C}\left( t \right)+{fn}_{6}$where $f_{1}$,$f_{2}$,$f_{3}$,$f_{4}, f$,$f_{6}$are constants,$\frac{\partial V_{h}^{C}}{\partial G^{C}}=f_{1}$, $\frac{\partial V_{h}^{C}}{\partial W^{C}}=f_{2}$, , $\frac{\text{∂}V_{o}^{C}}{\text{∂}\text{G}^{\text{C}}}=f_{4}$, $\frac{\text{∂}V_{o}^{C}}{\text{∂}\text{W}^{\text{C}}}=f_{5}$, then yields:

$$\begin{aligned} &\rho\left( f_{1}G^{C}\left( t \right)+f_{2}W^{C}\left( t \right)+f_{3} \right) \\ & =\left\{ \begin{aligned} \pi_{h}&\left( \alpha G^{C}\left( t \right)+\beta W^{C}\left( t \right) \right)-\frac{{\tau_{1}}^{2}}{\text{2}\mu_{1}}\left( \frac{\partial V_{h}^{C}}{\partial G^{C}} \right)^{\text{2}}-\frac{{\tau_{2}}^{2}}{\text{2}\mu_{2}}\left( \frac{\partial V_{h}^{C}}{\partial W^{C}} \right)^{\text{2}}e^{2\delta d_{h}} \\ +&\frac{{\tau_{1}}^{2}}{\mu_{1}}\left( \frac{\partial V_{h}^{C}}{\partial G^{C}} \right)^{\text{2}}-\gamma{\frac{\partial V_{h}^{C}}{\partial G^{C}}G}^{C}\left( t \right)+\eta\frac{\partial V_{h}^{C}}{\partial W^{C}}G^{C}\left( t \right) \\ +&\frac{{\tau_{2}}^{2}}{\mu_{2}}\left( \frac{\partial V_{h}^{C}}{\partial W^{C}} \right)^{\text{2}}e^{2\delta d_{h}}+\frac{{\tau_{3}}^{2}}{\mu_{3}}\frac{\partial V_{h}^{C}}{\partial W^{C}}\frac{\partial V_{o}^{C}}{\partial W^{C}}e^{2\delta d_{o}}+\delta\frac{\partial V_{h}^{C}}{\partial W^{C}}W^{C}\left( t \right) \end{aligned} \right\}\#\left( A.15 \right) \end{aligned}$$

$$\begin{aligned} &\rho\left( f_{4}G^{C}\left( t \right)+f_{5}W^{C}\left( t \right)+{fn}_{6} \right) \\ & =\left\{ \begin{aligned} \pi_{o}&\left( \alpha G^{C}\left( t \right)+\beta W^{C}\left( t \right) \right)-\frac{{\tau_{3}}^{2}}{\text{2}\mu_{3}}\left( \frac{\text{∂}V_{o}^{C}}{\text{∂}\text{W}^{\text{C}}} \right)^{\text{2}}e^{2\delta d_{o}} \\ +&\frac{{\tau_{1}}^{2}}{\mu_{1}}\frac{\text{∂}V_{h}^{C}}{\text{∂}\text{G}^{\text{C}}}\frac{\text{∂}V_{o}^{C}}{\text{∂}\text{G}^{\text{C}}}-\gamma{\frac{\partial V_{o}^{C}}{\partial G^{C}}G}^{C}\left( t \right)+\eta\frac{\partial V_{o}^{C}}{\partial W^{C}}G^{C}\left( t \right) \\ +&\frac{{\tau_{2}}^{2}}{\mu_{2}}\frac{\partial V_{h}^{C}}{\partial W^{C}}\frac{\partial V_{o}^{C}}{\partial W^{C}}e^{2\delta d_{h}}+\frac{{\tau_{3}}^{2}}{\mu_{3}}\left( \frac{\text{∂}V_{O}^{C}}{\text{∂}\text{W}^{\text{C}}} \right)^{\text{2}}e^{2\delta d_{o}}-\delta{\frac{\text{∂}V_{o}^{C}}{\text{∂}\text{W}^{\text{C}}}W}^{C}\left( t \right) \end{aligned} \right\}\#\left( A.16 \right) \end{aligned}$$

From Eq. (A.15) and (A.16), yields

$$\begin{aligned} \left\{ \begin{aligned} f_{1}&=\frac{\pi_{h}\left( \alpha\left( \rho+\delta\right)+\beta\eta\right)}{\left( \rho+\gamma\right)\left( \rho+\delta\right)} \\ f_{2}&=\frac{\beta\pi_{h}}{\left( \rho+\delta\right)} \\ f_{3}&=\frac{{\pi_{h}}^{2}\left( \alpha\left( \rho+\delta\right)+\beta\eta\right)^{2}}{2\rho{\left( \rho+\delta\right)^{2}\left( \rho+\gamma\right)}^{2}}\frac{{\tau_{1}}^{2}}{\mu_{1}}+\frac{{\pi_{h}}^{2}\beta^{2}}{2\rho({\rho+\delta)}^{2}}\frac{{\tau_{2}}^{2}}{\mu_{2}}e^{2\delta d_{h}}+\frac{\pi_{h}\pi_{o}\beta^{2}}{\rho({\rho+\delta)}^{2}}\frac{{\tau_{3}}^{2}}{\mu_{3}}e^{2\delta d_{o}} \end{aligned} \right.\#\left( A.17 \right) \end{aligned}$$

And

$$\begin{aligned} \left\{ \begin{aligned} f_{4}&=\frac{\pi_{o}\left( \alpha\left( \rho+\delta\right)+\beta\eta\right)}{\left( \rho+\gamma\right)\left( \rho+\delta\right)} \\ f_{5}&=\frac{\beta\pi_{o}}{\left( \rho+\delta\right)} \\ f_{6}&=\frac{{\pi_{o}}^{2}\beta^{2}}{2\rho({\rho+\delta)}^{2}}\frac{{\tau_{3}}^{2}}{\mu_{3}}e^{2\delta d_{o}}+\frac{\pi_{h}\pi_{o}\beta^{2}}{\rho({\rho+\delta)}^{2}}\frac{{\tau_{2}}^{2}}{\mu_{2}}e^{2\delta d_{h}}+\frac{\pi_{h}\pi_{o}\left( \alpha\left( \rho+\delta\right)+\beta\eta\right)^{2}}{\rho{\left( \rho+\delta\right)^{2}\left( \rho+\gamma\right)}^{2}}\frac{{\tau_{1}}^{2}}{\mu_{1}} \end{aligned} \right.\#\left( A.18 \right) \end{aligned}$$

Substituting, $\frac{\partial V_{h}^{C}}{\partial G^{C}}=f_{1}$, $\frac{\partial V_{h}^{C}}{\partial W^{C}}=f_{2}$, $f_{3}$ into Eq. (A.12) and$\frac{\text{∂}V_{o}^{C}}{\text{∂}\text{G}^{\text{C}}}=f_{4}$, $\frac{\text{∂}V_{o}^{C}}{\text{∂}\text{W}^{\text{C}}}=f_{5}$,$f_{6}$ into Eq. (A.12) , we can obtain Proposition 2.

**Proof for proposition 5**

For any $G^{S}$(t) > 0, $W^{S}$(t) > 0 the maximal profits of the GSMHE and the OTA at time t in the Hamilton-Jacobi-Bellman equation satisfy the following equations:

$$\begin{aligned} \rho V_{h}^{S}=\max_{S_{h}^{S},E_{h}^{S}} \left\{ \begin{aligned} &\pi_{h}\left( \alpha G^{S}\left( t \right)+\beta W^{S}\left( t \right) \right)-\frac{\mu_{1}}{\text{2}}{S_{h}^{S}}^{\text{2}}-\frac{\mu_{2}}{\text{2}}{{\left( 1-\Phi\right)E}_{h}^{S}}^{\text{2}} \\ &+\frac{\partial V_{h}^{S}}{\partial G^{S}}\left( \tau_{1}S_{h}^{S}\left( t \right)-\gamma G^{S}\left( t \right) \right) \\ &+\frac{\partial V_{h}^{S}}{\partial W^{S}}\left( \eta G^{S}\left( t \right)+\tau_{2}E_{o}^{S}\left( t-d_{h} \right)+\tau_{3}E_{o}^{S}\left( t-d_{o} \right)-\delta W^{S}\left( t \right) \right) \end{aligned} \right\}\#\left( A.19 \right) \end{aligned}$$

$$\begin{aligned} \rho V_{o}^{S}=\max_{E_{o}^{S},\Phi_{,}} \left\{ \begin{aligned} &\pi_{0}\left( \alpha G^{S}\left( t \right)+\beta W^{S}\left( t \right) \right)-\frac{\mu_{3}}{\text{2}}{E_{o}^{S}}^{\text{2}}-\frac{\mu_{2}}{\text{2}}{{\Phi E}_{h}^{S}}^{\text{2}} \\ &+\frac{\partial V_{o}^{S}}{\partial G^{S}}\left( \tau_{1}S_{h}^{S}\left( t \right)-\gamma G^{S}\left( t \right) \right) \\ &+\frac{\partial V_{o}^{S}}{\partial W^{S}}\left( \eta G^{S}\left( t \right)+\tau_{2}E_{o}^{S}\left( t-d_{h} \right)+\tau_{3}E_{o}^{S}\left( t-d_{o} \right)-\delta W^{S}\left( t \right) \right) \end{aligned} \right\}\#\left( A.20 \right) \end{aligned}$$

According to studies of *Basin and Rodriguez-Gonzalez (2006)* , *Sun, et al. (2020)* ,$\frac{\partial E_{o}(t-d_{o})}{\partial E_{o}(t)}=e^{\delta d_{o}}$,$\frac{\partial E_{h}(t-d_{h})}{\partial E_{h}(t)}=e^{\delta d_{h}}$.

From Eq. (A.19) and (A.20), we can obtain:

$$\begin{aligned} \left\{ \begin{aligned} S_{h}^{S*}&=\frac{\partial V_{h}^{S}}{\partial G^{S}}\frac{\tau_{1}}{\mu_{1}} \\ E_{o}^{S*}&=\frac{\partial V_{h}^{S}}{\partial W^{S}\left( 1-\Phi\right)}\frac{\tau_{2}}{\mu_{2}}e^{\delta d_{h}} \\ E_{o}^{S*}&=\frac{\partial V_{o}^{S}}{\partial W^{S}}\frac{\tau_{3}}{\mu_{3}}e^{\delta d_{o}} \\ \Phi^{*}&=\left\{ \begin{matrix} \frac{2\pi_{o}-\pi_{h}}{2\pi_{o}+\pi_{h}}, & \pi_{o}\geq\frac{\pi_{h}}{2} \\ 0, & \pi_{o}<\frac{\pi_{h}}{2} \end{matrix} \right. \end{aligned} \right.\#\left( A.21 \right) \end{aligned}$$

Substituting Eq. (A.21) into the Eq. (A.19) and (A.20), yields

$$\begin{aligned} \rho V_{h}^{S}=\left\{ \begin{aligned} &\pi_{h}\left( \alpha G^{S}\left( t \right)+\beta W^{S}\left( t \right) \right)-\frac{{\tau_{1}}^{2}}{\text{2}\mu_{1}}\left( \frac{\partial V_{h}^{S}}{\partial G^{S}} \right)^{\text{2}}-\frac{{\tau_{2}}^{2}}{\text{2}\mu_{2}\left( 1-\Phi\right)}\left( \frac{\text{∂}V_{h}^{S}}{\text{∂}\text{W}^{\text{S}}} \right)^{\text{2}}e^{2\delta d_{h}} \\ &+\frac{{\tau_{1}}^{2}}{\mu_{1}}\left( \frac{\partial V_{h}^{S}}{\partial G^{S}} \right)^{\text{2}}-\gamma{\frac{\partial V_{h}^{S}}{\partial G^{S}}G}^{S}\left( t \right)+\eta\frac{\partial V_{h}^{S}}{\partial W^{S}}G^{C}\left( t \right) \\ &+\frac{{\tau_{2}}^{2}}{\mu_{2}\left( 1-\Phi\right)}\left( \frac{\partial V_{h}^{S}}{\partial W^{S}} \right)^{\text{2}}e^{2\delta d_{h}}+\frac{{\tau_{3}}^{2}}{\mu_{3}}\frac{\partial V_{h}^{S}}{\partial W^{S}}\frac{\partial V_{o}^{S}}{\partial W^{S}}e^{2\delta d_{o}}-\delta\frac{\partial V_{h}^{S}}{\partial W^{S}}W^{S}\left( t \right) \end{aligned} \right\}\#\left( A.22 \right) \end{aligned}$$

$$\begin{aligned} \rho V_{o}^{S}=\left\{ \begin{aligned} &\pi_{o}\cdot\left( \alpha G^{S}\left( t \right)+\beta W^{S}\left( t \right) \right)-\frac{\Phi{\tau_{2}}^{2}}{\text{2}\mu_{2}\left( 1-\Phi\right)^{2}}\left( \frac{\text{∂}V_{h}^{S}}{\text{∂}\text{W}^{\text{S}}} \right)^{\text{2}}e^{2\delta d_{h}} \\ &-\frac{{\tau_{3}}^{2}}{\text{2}\mu_{3}}\left( \frac{\text{∂}V_{o}^{S}}{\text{∂}\text{W}^{\text{S}}} \right)^{\text{2}}e^{2\delta d_{o}}+\frac{{\tau_{1}}^{2}}{\mu_{1}}\frac{\text{∂}V_{h}^{S}}{\text{∂}\text{G}^{\text{S}}}\frac{\text{∂}V_{o}^{S}}{\text{∂}\text{G}^{\text{S}}}-\gamma{\frac{\partial V_{o}^{S}}{\partial G^{S}}G}^{C}\left( t \right)+\eta\frac{\partial V_{o}^{S}}{\partial W^{S}}G^{S}\left( t \right) \\ &+\frac{{\tau_{2}}^{2}}{\mu_{2}\left( 1-\Phi\right)}\frac{\partial V_{h}^{S}}{\partial W^{S}}\frac{\partial V_{o}^{S}}{\partial W^{S}}e^{2\delta d_{h}}+\frac{{\tau_{3}}^{2}}{\mu_{3}}\left( \frac{\text{∂}V_{o}^{S}}{\text{∂}\text{W}^{\text{S}}} \right)^{\text{2}}e^{2\delta d_{o}}-\delta{\frac{\text{∂}V_{o}^{S}}{\text{∂}\text{W}^{\text{S}}}W}^{S}\left( t \right) \end{aligned} \right\}\#\left( A.23 \right) \end{aligned}$$

It can be seen from Eq. (A22) and (A23) that the solution of the HJB equation is a linear function of G(t), W(t) , then we set $V_{h}^{S}=h_{1}G^{S}\left( t \right)+h_{2}W^{S}\left( t \right)+h_{3}$, $V_{o}^{S}=h_{4}G^{S}\left( t \right)+h_{5}W^{S}\left( t \right)+h_{6}$where $h_{1}$,$h_{2}$,$h_{3}$,$h_{4}, h$,$h_{6}$are constants, $\frac{\partial V_{h}^{C}}{\partial G^{C}}=f_{1}$, $\frac{\partial V_{h}^{C}}{\partial W^{C}}=f_{2}$, $\frac{\text{∂}V_{o}^{C}}{\text{∂}\text{G}^{\text{C}}}=f_{4}$, $\frac{\text{∂}V_{o}^{C}}{\text{∂}\text{W}^{\text{C}}}=f_{5}$, then yields:

$$\begin{aligned} &\rho\left( h_{1}G^{S}\left( t \right)+h_{2}W^{S}\left( t \right)+h_{3} \right) \\ &=\left\{ \begin{aligned} \pi_{h}&\left( \alpha G^{S}\left( t \right)+\beta W^{S}\left( t \right) \right)-\frac{{\tau_{1}}^{2}}{\text{2}\mu_{1}}\left( \frac{\partial V_{h}^{S}}{\partial G^{S}} \right)^{\text{2}}-\frac{{\tau_{2}}^{2}}{\text{2}\mu_{2}\left( 1-\Phi\right)}\left( \frac{\text{∂}V_{h}^{S}}{\text{∂}\text{W}^{\text{S}}} \right)^{\text{2}}e^{2\delta d_{h}} \\ +&\frac{{\tau_{1}}^{2}}{\mu_{1}}\left( \frac{\partial V_{h}^{S}}{\partial G^{S}} \right)^{\text{2}}-\gamma{\frac{\partial V_{h}^{S}}{\partial G^{S}}G}^{S}\left( t \right)+\eta\frac{\partial V_{h}^{S}}{\partial W^{S}}G^{C}\left( t \right) \\ +&\frac{{\tau_{2}}^{2}}{\mu_{2}\left( 1-\Phi\right)}\left( \frac{\partial V_{h}^{S}}{\partial W^{S}} \right)^{\text{2}}e^{2\delta d_{h}}+\frac{{\tau_{3}}^{2}}{\mu_{3}}\frac{\partial V_{h}^{S}}{\partial W^{S}}\frac{\partial V_{o}^{S}}{\partial W^{S}}e^{2\delta d_{o}}-\delta\frac{\partial V_{h}^{S}}{\partial W^{S}}W^{S}\left( t \right) \end{aligned} \right\}\#\left( A.24 \right) \end{aligned}$$

$$\begin{aligned} &\rho\left( h_{4}G^{S}\left( t \right)+h_{5}W^{S}\left( t \right)+h_{6} \right) \\ &=\left\{ \begin{aligned} &\pi_{o}\cdot\left( \alpha G^{S}\left( t \right)+\beta W^{S}\left( t \right) \right)-\frac{\Phi{\tau_{2}}^{2}}{\text{2}\mu_{2}\left( 1-\Phi\right)^{2}}\left( \frac{\text{∂}V_{h}^{S}}{\text{∂}\text{W}^{\text{S}}} \right)^{\text{2}}e^{2\delta d_{h}} \\ &-\frac{{\tau_{3}}^{2}}{\text{2}\mu_{3}}\left( \frac{\text{∂}V_{o}^{S}}{\text{∂}\text{W}^{\text{S}}} \right)^{\text{2}}e^{2\delta d_{o}}+\frac{{\tau_{1}}^{2}}{\mu_{1}}\frac{\text{∂}V_{h}^{S}}{\text{∂}\text{G}^{\text{S}}}\frac{\text{∂}V_{o}^{S}}{\text{∂}\text{G}^{\text{S}}}-\gamma{\frac{\partial V_{o}^{S}}{\partial G^{S}}G}^{C}\left( t \right)+\eta\frac{\partial V_{o}^{S}}{\partial W^{S}}G^{S}\left( t \right) \\ &+\frac{{\tau_{2}}^{2}}{\mu_{2}\left( 1-\Phi\right)}\frac{\partial V_{h}^{S}}{\partial W^{S}}\frac{\partial V_{o}^{S}}{\partial W^{S}}e^{2\delta d_{h}}+\frac{{\tau_{3}}^{2}}{\mu_{3}}\left( \frac{\text{∂}V_{o}^{S}}{\text{∂}\text{W}^{\text{S}}} \right)^{\text{2}}e^{2\delta d_{o}}-\delta{\frac{\text{∂}V_{o}^{S}}{\text{∂}\text{W}^{\text{S}}}W}^{S}\left( t \right) \end{aligned} \right\}\#\left( A.25 \right) \end{aligned}$$

From Eq. (A24) and (A.25), yields

$$\begin{aligned} \left\{ \begin{aligned} h_{1}&=\frac{\pi_{h}\left( \alpha\left( \rho+\delta\right)+\beta\eta\right)}{\left( \rho+\gamma\right)\left( \rho+\delta\right)} \\ h_{2}&=\frac{\beta\pi_{h}}{\left( \rho+\delta\right)} \\ h_{3}&=\frac{{\pi_{h}}^{2}\left( \alpha\left( \rho+\delta\right)+\beta\eta\right)^{2}}{2\rho{\left( \rho+\delta\right)^{2}\left( \rho+\gamma\right)}^{2}}\frac{{\tau_{1}}^{2}}{\mu_{1}}+\frac{{\pi_{h}}^{2}\beta^{2}}{2\rho({\rho+\delta)}^{2}(1-\Phi)}\frac{{\tau_{2}}^{2}}{\mu_{2}}e^{2\delta d_{h}} \\ &+\frac{\pi_{h}\pi_{o}\beta^{2}}{\rho({\rho+\delta)}^{2}}\frac{{\tau_{3}}^{2}}{\mu_{3}}e^{2\delta d_{o}} \end{aligned} \right.\left( A.26 \right) \end{aligned}$$

And

$$\begin{aligned} \left\{ \begin{aligned} h_{4}&=\frac{\pi_{o}\left( \alpha\left( \rho+\delta\right)+\beta\eta\right)}{\left( \rho+\gamma\right)\left( \rho+\delta\right)} \\ h_{5}&=\frac{\beta\pi_{o}}{\left( \rho+\delta\right)} \\ h_{6}&=\frac{{\pi_{o}}^{2}\beta^{2}}{2\rho({\rho+\delta)}^{2}}\frac{{\tau_{3}}^{2}}{\mu_{3}}e^{2\delta d_{o}}+\frac{\pi_{h}\beta^{2}}{2\rho({\rho+\delta)}^{2}}\frac{{\tau_{2}}^{2}}{\mu_{2}}e^{2\delta d_{h}}\left( \frac{{2\pi}_{o}\left( 1-\Phi\right)-\pi_{h}\Phi}{({1-\Phi)}^{2}} \right) \\ & +\frac{\pi_{h}\pi_{o}\left( \alpha\left( \rho+\delta\right)+\beta\eta\right)^{2}}{\rho{\left( \rho+\delta\right)^{2}\left( \rho+\gamma\right)}^{2}}\frac{{\tau_{1}}^{2}}{\mu_{1}} \end{aligned} \right.\#\left( A.27 \right) \end{aligned}$$

Substituting, $\frac{\partial V_{h}^{C}}{\partial G^{C}}=f_{1}$, $\frac{\partial V_{h}^{C}}{\partial W^{C}}=f_{2}$, $f_{3}$ into Eq. (A.12) and $\frac{\text{∂}V_{o}^{C}}{\text{∂}\text{G}^{\text{C}}}=f_{4}$, $\frac{\text{∂}V_{o}^{C}}{\text{∂}\text{W}^{\text{C}}}=f_{5}$,$f_{6}$ into Eq. (A.21) we can obtain Proposition5.

**Proof for Corollary 1**

$E_{h}^{S*}-E_{h}^{C*}$=$\frac{\Phi\pi_{h}\beta}{\left( 1-\Phi\right)\left( \rho+\delta\right)}\frac{\tau_{2}}{\mu_{2}}e^{\delta d_{h}}$>0

**Proof for Corollary 2**

$W_{\infty}^{C*}-W_{\infty}^{O*}$=$\frac{\pi_{h}\beta}{\left( \rho+\delta\right)\delta}\cdot\frac{{\tau_{2}}^{2}}{\mu_{2}}{\cdot e}^{\delta d_{h}}$>0

$W_{\infty}^{S*}-W_{\infty}^{C*}$=$\frac{\pi_{h}\beta\Phi}{\left( \rho+\delta\right)\left( 1-\Phi\right)\delta}\frac{{\tau_{2}}^{2}}{\mu_{2}}e^{\delta d_{h}}$>0

**Proof for Corollary 3**

$J_{h}^{C*}-J_{h}^{O*}$=$\frac{{\pi_{h}}^{2}\beta^{2}}{\rho\left( \rho+\delta\right)^{2}}\frac{{\tau_{2}}^{2}}{\mu_{2}}{(e}^{\delta d_{h}}-\frac{e^{2\delta d_{h}}}{2})$ when $d_{h}<\frac{\ln^{2}}{\delta}$ , $J_{h}^{O*}<J_{h}^{C*}$. when$d_{h}>\frac{\ln^{2}}{\delta}$, $J_{h}^{C*}<J_{h}^{O*}$.

$J_{h}^{S*}-J_{h}^{C*}$=$\frac{{\pi_{h}}^{2}\beta^{2}}{\rho\left( \rho+\delta\right)^{2}\left( 1-\Phi\right)}\frac{{\tau_{2}}^{2}}{\mu_{2}}{(e}^{\delta d_{h}}-\frac{e^{2\delta d_{h}}}{2})$ when $d_{h}<\frac{\ln^{2}}{\delta}$ , $J_{h}^{C*}<J_{h}^{S*}$, when $d_{h}>\frac{\ln^{2}}{\delta}$, $J_{h}^{S*}<J_{h}^{C*}$.

**Proof for Corollary 4**

$J_{o}^{C*}-J_{o}^{O*}$=$\frac{\pi_{o}\pi_{h}\beta^{2}}{\rho\left( \rho+\delta\right)^{2}}\frac{{\tau_{2}}^{2}}{\mu_{2}}e^{\delta d_{h}}$>0

$J_{o}^{S*}-J_{o}^{C*}$=$\frac{\pi_{h}\beta^{2}e^{\delta d_{h}}\Phi}{\rho\left( \rho+\delta\right)^{2}\left( 1-\Phi\right)}\frac{{\tau_{2}}^{2}}{\mu_{2}}\left( \pi_{o}-\frac{\pi_{h}}{2\left( 1-\Phi\right)}e^{\delta d_{h}} \right)$

When $d_{h}<\frac{\ln^{\frac{4\pi_{o}}{2\pi_{o}+\pi_{h}}}}{\delta}$, $J_{o}^{C*}$<$J_{o}^{S*}$, $d_{h}>\frac{\ln^{\frac{4\pi_{o}}{2\pi_{o}+\pi_{h}}}}{\delta}$, $J_{o}^{S*}$<$J_{o}^{C*}$

$J_{o}^{S*}-J_{o}^{O*}$=$\frac{\pi_{h}\beta^{2}e^{\delta d_{h}}}{\rho\left( \rho+\delta\right)^{2}\left( 1-\Phi\right)}\frac{{\tau_{2}}^{2}}{\mu_{2}}\left( \pi_{o}-\frac{\pi_{h} \Phi}{2\left( 1-\Phi\right)}e^{\delta d_{h}} \right)$

When $d_{h}<\frac{\ln^{\frac{4\pi_{o}}{2\pi_{o}{-\pi}_{h}}}}{\delta}$, $J_{o}^{O*}$<$J_{o}^{S*}$, $d_{h}>\frac{\ln^{\frac{4\pi_{o}}{2\pi_{o}{-\pi}_{h}}}}{\delta}$, $J_{o}^{S*}$<$J_{o}^{O*}$

**Proofs of Corollary 5**

(i) $\frac{\partial S_{h}^{\star}}{\partial\tau_{1}}$= $\frac{\pi_{h}\left( \alpha\left( \rho+\delta\right)+\beta\eta\right)}{\left( \rho+\gamma\right)\left( \rho+\delta\right)\mu_{1}}$>0, $\frac{\partial S_{h}^{\star}}{\partial\alpha}$=$\frac{\pi_{h} \tau_{1}}{\left( \rho+\gamma\right)\mu_{1}}$>0, $\frac{\partial S_{h}^{\star}}{\partial\eta}=\frac{\pi_{h}\beta\eta\tau_{1}}{\left( \rho+\gamma\right)\left( \rho+\delta\right)\mu_{1}}$>0,
 $\frac{\partial S_{h}^{\star}}{\partial\beta}=\frac{\pi_{h}\beta\tau_{1}}{\left( \rho+\gamma\right)\left( \rho+\delta\right)\mu_{1}}$>0, $\frac{\partial S_{h}^{\star}}{\partial\gamma}=-\frac{\pi_{h}\left( \alpha\left( \rho+\delta\right)+\beta\eta\right)\tau_{1}}{\left( \rho+\gamma\right)^{2}\left( \rho+\delta\right)\mu_{1}}<0$,
 $\frac{\partial S_{h}^{\star}}{\partial\delta}=-\frac{\pi_{h}\beta\eta\tau_{1}}{\left( \rho+\gamma\right)\left( \rho+\delta\right)^{2}\mu_{1}}<0$

(ii) $\frac{\partial E_{h}^{C\star}}{\partial\tau_{2}}=\frac{\pi_{h}\beta}{\left( \rho+\delta\right)\mu_{2}}e^{\delta d_{h}}>0$, $\frac{\partial E_{h}^{S\star}}{\partial\tau_{2}}=\frac{\pi_{h}\beta}{\left( \rho+\delta\right)\left( 1-\Phi\right)\mu_{2}}e^{\delta d_{h}}>0$,

$\frac{\partial E_{h}^{C\star}}{\partial\beta}=\frac{\pi_{h}\tau_{2}}{\left( \rho+\delta\right)\mu_{2}}e^{\delta d_{h}}>0$, $\frac{\partial E_{h}^{C\star}}{\partial\beta}=\frac{\pi_{h}\tau_{2}}{\left( \rho+\delta\right)\left( 1-\Phi\right)\mu_{2}}e^{\delta d_{h}}>0$,

$\frac{\partial E_{o}^{\star}}{\partial\tau_{3}}=\frac{\pi_{o}\beta}{\left( \rho+\delta\right)\mu_{3}}e^{\delta d_{o}}>0$, $\frac{\partial E_{o}^{\star}}{\partial\beta}=\frac{\pi_{h}\tau_{3}}{\left( \rho+\delta\right)\mu_{3}}e^{\delta d_{o}}>0$

(iii) $\frac{\partial G_{\infty}^{*}}{\partial\tau_{1}}=\frac{2\pi_{h}\left( \alpha\left( \rho+\delta\right)+\beta\eta\right)}{\left( \rho+\gamma\right)\left( \rho+\delta\right)\gamma}\cdot\frac{\tau_{1}}{\mu_{1}}>0$，$\frac{\partial G_{\infty}^{*}}{\partial\alpha}=\frac{\pi_{h}}{\left( \rho+\gamma\right)\gamma}\frac{{\tau_{1}}^{2}}{\mu_{1}}>0$，

$\frac{\partial G_{\infty}^{*}}{\partial\eta}=\frac{\pi_{h} \beta}{\left( \rho+\delta\right)\left( \rho+\gamma\right)\gamma}\frac{{\tau_{1}}^{2}}{\mu_{1}}>0$， $\frac{\partial G_{\infty}^{*}}{\partial\beta}=\frac{\pi_{h} \eta}{\left( \rho+\delta\right)\left( \rho+\gamma\right)\gamma}\frac{{\tau_{1}}^{2}}{\mu_{1}}>0$

$\frac{\partial G_{\infty}^{*}}{\partial\gamma}$=$-\frac{\pi_{h}\left( \alpha\left( \rho+\delta\right)+\beta\eta\right)}{\left( \rho+\gamma\right)\left( \rho+\delta\right)\gamma}\frac{{\tau_{1}}^{2}}{\mu_{1}}\left（ \frac{1}{\rho+\gamma}+\frac{1}{\gamma} \right）$<0

$\frac{\partial G_{\infty}^{*}}{\partial\delta}= -\frac{\pi_{h}\beta\eta}{\left( \rho+\gamma\right)\left( \rho+\delta\right)^{2}\gamma}\frac{{\tau_{1}}^{2}}{\mu_{1}}<0$

(iv$) \frac{\partial W_{\infty}^{*}}{\partial\tau_{1}}=\frac{2\pi_{h}\left( \alpha\left( \rho+\delta\right)+\beta\eta\right)\eta}{\left( \rho+\gamma\right)\left( \rho+\delta\right)\gamma\delta}\cdot\frac{\tau_{1}}{\mu_{1}}>0$, $\frac{\partial W_{\infty}^{*}}{\partial\tau_{2}}=\frac{2\pi_{h}\beta}{\left( \rho+\delta\right)\delta}\cdot\frac{\tau_{2}}{\mu_{2}}{\cdot e}^{\delta d_{h}}>0$,

$\frac{\partial W_{\infty}^{*}}{\partial\tau_{3}}=\frac{2\pi_{o}\beta}{\left( \rho+\delta\right)\delta}\cdot\frac{\tau_{3}}{\mu_{3}}{\cdot e}^{\delta d_{o}}>0$, $\frac{\partial W_{\infty}^{*}}{\partial\alpha}=\frac{\pi_{h} \eta}{\left( \rho+\gamma\right)\gamma\delta}\frac{{\tau_{1}}^{2}}{\mu_{1}}>0$,

$\frac{\partial W_{\infty}^{*}}{\partial\eta}=\frac{\pi_{h}}{\left( \rho+\delta\right)\left( \rho+\gamma\right)\gamma\delta}\frac{{\tau_{1}}^{2}}{\mu_{1}}\left（ 2\beta\eta+\alpha\left( \rho+\delta\right) \right）>0$

$\frac{\partial W_{\infty}^{*}}{\partial\beta}=\frac{\pi_{h} \eta^{2}}{\left( \rho+\delta\right)\left( \rho+\gamma\right)\gamma\delta}\frac{{\tau_{1}}^{2}}{\mu_{1}}+\frac{\pi_{h}}{\left( \rho+\delta\right)\delta}\cdot\frac{{\tau_{2}}^{2}}{\mu_{2}}{\cdot e}^{\delta d_{h}}+\frac{\pi_{o}}{\left( \rho+\delta\right)\delta}\cdot\frac{{\tau_{3}}^{2}}{\mu_{3}}{\cdot e}^{\delta d_{o}}>0$

**Proofs of Corollary 6**

(i) $\frac{\partial E_{h}^{C\star}}{\partial d_{h}}$= $\frac{\pi_{h}\beta\delta}{\left( \rho+\delta\right)}\frac{\tau_{2}}{\mu_{2}}e^{\delta d_{h}}$>0, $\frac{\partial E_{h}^{S\star}}{\partial d_{h}}=\frac{\pi_{h}\beta\delta}{\left( \rho+\delta\right)\left( 1-\Phi\right)}\frac{\tau_{2}}{\mu_{2}}e^{\delta d_{h}}$>0

$\frac{\partial E_{o}^{\star}}{\partial d_{o}}$= $\frac{\pi_{o}\beta\delta}{\left( \rho+\delta\right)}\frac{\tau_{3}}{\mu_{3}}e^{\delta d_{o}}$>0

(ii) $\frac{\partial W_{\infty}^{C*}}{\partial d_{h}}=\frac{\pi_{h}\beta}{\left( \rho+\delta\right)}\cdot\frac{{\tau_{2}}^{2}}{\mu_{2}}{\cdot e}^{\delta d_{h}}>0$,$\frac{\partial W_{\infty}^{S*}}{\partial d_{h}}=\frac{\pi_{h}\beta}{\left( 1-\Phi\right)\left( \rho+\delta\right)}\cdot\frac{{\tau_{2}}^{2}}{\mu_{2}}{\cdot e}^{\delta d_{h}}>0$
 $\frac{\partial W_{\infty}^{O*}}{\partial d_{o}}$=$\frac{\partial W_{\infty}^{C*}}{\partial d_{o}}=$ $\frac{\partial W_{\infty}^{S*}}{\partial d_{o}}=\frac{\pi_{o}\beta}{\left( \rho+\delta\right)}\cdot\frac{{\tau_{3}}^{2}}{\mu_{3}}{\cdot e}^{\delta d_{o}}>0$

$\left( = 2 \backslash* roman \mathrm{ii}i \right)\frac{\partial J_{h}^{C^{\star}}}{\partial d_{h}}$=$\frac{{\pi_{h}}^{2}\beta^{2}\delta}{\rho\left( \rho+\delta\right)^{2}}\frac{{\tau_{2}}^{2}}{\mu_{2}}e^{\delta d_{h}}(1-e^{\delta d_{h}})$<0

$\frac{\partial J_{h}^{S^{\star}}}{\partial d_{h}}$ = $\frac{{\pi_{h}}^{2}\beta^{2}\delta}{\rho{\left( 1-\Phi\right)\left( \rho+\delta\right)}^{2}}\frac{{\tau_{2}}^{2}}{\mu_{2}}e^{\delta d_{h}}(1-e^{\delta d_{h}})$<0

$\frac{\partial J_{o}^{C^{\star}}}{\partial d_{h}}$ = $\frac{\pi_{o}\pi_{h}\beta^{2}\delta}{\rho\left( \rho+\delta\right)^{2}}\frac{{\tau_{2}}^{2}}{\mu_{2}}e^{\delta d_{h}}$>0

$\frac{\partial J_{o}^{S^{\star}}}{\partial d_{h}}$ = $\frac{\pi_{h}\beta^{2}\delta}{\rho{\left( 1-\Phi\right)\left( \rho+\delta\right)}^{2}}\frac{{\tau_{2}}^{2}}{\mu_{2}}e^{\delta d_{h}}\left( \pi_{o}-\frac{\pi_{h}\Phi}{\left( 1-\Phi\right)}e^{\delta d_{h}} \right)$ $=\frac{\pi_{h}\beta^{2}\delta}{\rho{\left( 1-\Phi\right)\left( \rho+\delta\right)}^{2}}\frac{{\tau_{2}}^{2}}{\mu_{2}}e^{\delta d_{h}}\left( \pi_{o}-\frac{2\pi_{o}-\pi_{h}}{2}e^{\delta d_{h}} \right)$ when $d_{h}>\frac{\ln^{\frac{2\pi_{o}}{2\pi_{o}-\pi_{h}}}}{\delta}$, $\frac{\partial J_{o}^{S^{\star}}}{\partial d_{h}}$<0, when $d_{h}<\frac{\ln^{\frac{2\pi_{o}}{2\pi_{o}-\pi_{h}}}}{\delta}$, $\frac{\partial J_{o}^{S^{\star}}}{\partial d_{h}}$>0,

(iv$) \frac{\partial J_{h}^{O^{\star}}}{\partial d_{o}}$=$\frac{\partial J_{h}^{C^{\star}}}{\partial d_{o}}$=$\frac{\partial J_{h}^{S^{\star}}}{\partial d_{o}}$= $\frac{\pi_{o}\pi_{h}\beta^{2}\delta}{\rho\left( \rho+\delta\right)^{2}}\frac{{\tau_{3}}^{2}}{\mu_{3}}e^{\delta d_{o}}$>0

$\frac{\partial J_{o}^{O^{\star}}}{\partial d_{o}}$=$\frac{\partial J_{o}^{C^{\star}}}{\partial d_{o}}$=$\frac{\partial J_{o}^{S^{\star}}}{\partial d_{o}}$= $\frac{{\pi_{o}}^{2}\beta^{2}\delta}{\rho\left( \rho+\delta\right)^{2}}\frac{{\tau_{3}}^{2}}{\mu_{3}}e^{\delta d_{o}}(1-e^{\delta d_{o}})$
